# Factors predicting the therapeutic response to infliximab during maintenance therapy in Japanese patients with Crohn’s disease

**DOI:** 10.1371/journal.pone.0204632

**Published:** 2018-10-04

**Authors:** Katsuyoshi Matsuoka, Shunsuke Hamada, Mikiko Shimizu, Kosaku Nanki, Shinta Mizuno, Hiroki Kiyohara, Mari Arai, Shinya Sugimoto, Yasushi Iwao, Haruhiko Ogata, Tadakazu Hisamatsu, Makoto Naganuma, Takanori Kanai, Mayumi Mochizuki, Masayuki Hashiguchi

**Affiliations:** 1 Division of Gastroenterology and Hepatology, Department of Internal Medicine, Keio University School of Medicine, Shinanomachi, Shinjuku-ku, Tokyo, Japan; 2 Division for Evaluation and Analysis of Drug Information, Faculty of Pharmacy, Keio University, Shibakoen, Minato-ku, Tokyo, Japan; 3 Department of Hygienic Chemistry, Faculty of Pharmacy, Keio University, Shibakoen, Minato-ku, Tokyo, Japan; 4 Center for Preventive Medicine, Keio University School of Medicine, Shinanomachi, Shinjuku-ku, Tokyo, Japan; 5 Center for Diagnostic and Therapeutic Endoscopy, Keio University School of Medicine, Shinanomachi, Shinjuku-ku, Tokyo, Japan; Kurume University School of Medicine, JAPAN

## Abstract

Since anti-tumor necrosis factor (TNF)-α agents (TNF-α inhibitors) induce both clinical response and remission in patients with moderate to severe inflammatory bowel disease (IBD), the use of anti-TNF therapies has fundamentally changed the approach to treatment for patients with IBD. Infliximab (IFX) is a TNF-α inhibitor approved for the induction and remission of Crohn’s disease (CD). However, even among patients who initially demonstrate a clinical response to IFX therapy, secondary loss of response occurs, although the reason remains unknown. We therefore investigated predictive factors associated with the response to IFX in long-term maintenance treatment in Japanese CD patients. Eight types of single-nucleotide polymorphisms (SNPs) were investigated using the real-time PCR method, and patient characteristics were collected from the electronic medical records. The Crohn’s Disease Activity Index criteria were used as the response to IFX therapy. The observation period was 1 year after IFX had been administered for more than 1 year. Associations between the IFX response and patient characteristics were evaluated using the multivariate logistic regression model. We studied 121 unrelated adult Japanese with CD treated for more than 1 year with IFX as outpatients at Keio University Hospital from November 1, 2014 to November 30, 2015. Among them, 71 were classified as in remisson. In multivariate analysis, patients with the TNF-α 857C>T C/C genotype, shorter disease duration, without double dosing, and combination treatment with an immunomodulator had higher remisson rates than those with the C/T or T/T genotype, longer disease duration, with double dosing, and no combination treatment with an immunomodulator. The response to IFX in Japanese CD patients may therefore be predicted by these 4 characteristics in actual clinical practice.

## Introduction

Crohn’s disease (CD) is a chronic inflammatory condition of the gastrointestinal tract with repeated cycles of exacerbation and remission. Although the etiology of CD has not yet been revealed, it is considered to be caused by a breakdown of the intestinal immune system. The excessive production of tumor necrosis factor-α (TNF-α) in the intestinal mucosa driven by an aberrant immune reaction to luminal antigens was demonstrated to cause intestinal inflammation in patients with CD [1–3]. TNF-α further induces the production of proinflammatory cytokines such as interleukin-6, etc., thereby exacerbating inflammation. These observations led to the clinical application of anti-TNF-α antibody in the treatment of CD. Infliximab (IFX), a chimeric anti-TNF-α monoclonal antibody with the constant region of human IgG_1_ and the mouse-derived specific variable region against TNF-α, has been widely used for the treatment of CD [4,5]. IFX is thought to suppress TNF-α production by three mechanisms of action: 1) neutralization of the biological activity by binding soluble TNF-α; 2) dissociation of TNF-α bound to its receptor; and 3) killing cells that express membrane-bound TNF-α through antibody-dependent cellular cytotoxicity activity.

The development of this new drug has fundamentally changed the therapeutic strategy for CD. Mucosal healing rather than symptom relief has become the treatment goal because biologics can achieve mucosal healing at a higher rate than conventional agents such as 5-aminosalicylic acid (5-ASA), corticosteroids, and immunomodulators (IMs).

IFX is effective in treating patients with moderate to severe CD [3–7]; however, the therapeutic effect may be lost in some patients (i.e., loss of response; LOR) even with continuous IFX administration after the induction of remission. The rates of LOR are reported to be 50–54% in CD patients during 1 year of continuous IFX treatment [8,9]. LOR in patients receiving continuous IFX treatment is an important clinical problem, but the mechanisms are not yet fully understood. Previous studies reported that the production of antibody to infliximab (ATI) is a major cause of LOR. Furthermore, gene polymorphisms related to the mechanisms of action of IFX were found to contribute to individual differences in the response to IFX [10–21]. On the other hand, there is a controversial report that TNF and FcγRIIIA genotypes did not affect the response to IFX in this cohort of Greek patients with CD [22]. There are few reports on factors including gene polymorphisms that affect the response to IFX during maintenance therapy; in particular, only one such study was conducted in Japanese CD patients [23]. The purpose of this study was to clarify factors predicting the therapeutic effects of IFX during maintenance therapy in Japanese patients with CD.

## Patients and methods

### Patients

During the period from November 1, 2014, to November 30, 2015, we consecutively enrolled adult Japanese patients with CD treated with IFX for more than 1 year at Keio University Hospital. We prospectively followed the patients for 1 year after informed consent was obtained, collecting data on disease activity evaluated based on the Crohn’s Disease Activity Index (CDAI) [24] and C-reactive protein (CRP) levels at each visit for IFX administration.

Information on sex, height, body weight, age, disease duration (from the date of diagnosis to the date of informed consent), smoking history, IFX dose (5 mg/kg or 10 mg/kg), and concomitant medications (5-ASA, IM) was obtained from electronic medical records. 5-ASAs included mesalazine and salazosulfapyridine, and IMs included 6-mercaptopurine and azathioprine.

### Definition of therapeutic response

As an index of the therapeutic response to IFX, we combined the CDAI and CRP level. All CDAI scores and CRP levels evaluated during the 1-year follow-up were averaged in each patient to determine the final CDAI score and CRP level, respectively, because disease activity may transiently fluctuate during follow-up. Patients with CDAI <150 and CRP ≤0.3 were defined as in remission and the others as nonremission.

### Ethical considerations

The study protocol was approved by the Institutional Review Board of Keio University (approval nos. G151007-1, G161014-1, 20140445). All patients gave written informed consent for study participation prior to enrollment. This study was conducted in compliance with the Declaration of Helsinki.

### Blood and DNA extraction

For genetic analysis, 10 ml of peripheral blood was collected from each CD patient in a tube containing heparin sodium salt and stored at –80°C until DNA extraction. DNA was extracted using the Wizard Genomic DNA Purification Kit (Promega Corporation, Madison, WI, USA). Total genomic DNA was quantified and its purity and integrity were analyzed using Shimadzu BioSpec-nano (Shimadzu, Kyoto, Japan).

Additional blood sampling for genetic analysis was simultaneously performed at the first hospital visit after providing informed consent with routine blood sampling for clinical laboratory testing. After personal information linked to research subjects was anonymized, the samples were transported to Keio University, Faculty of Pharmacy, where genetic analysis was conducted.

### Genotyping of alleles of TNF, TNFR, and FCGR

Genotyping of alleles of TNF-α-238 G>A (rs361525), TNF-α-308 G>A (rs1800629), TNF-α-857 C>T (rs1799724), TNFR1 A>G (rs767455), TNFR2 G>A (rs976881), TNFR2 T>G (rs1061622), FCGR2A A>G (rs1801274), and FCGR3A T>G (rs396991) was performed using the TaqMan SNP Genotyping Assay from Applied Biosystems (Foster City, CA, USA) with fluorogenic binding probes. PCR amplification with the real-time PCR method was performed in 5 μL of reaction mixture including genomic DNA 4 ng, 0.0625 μL of 40 × TaqMan SNP Genotyping Assay Mix, and TaqMan Universal PCR Master Mix 2.5 μL. The reaction conditions for PCR were: pre-PCR for 30 s at 25°C; initial denaturation for 10 min at 95°C; 40 cycles at 92°C/15 s; annealing and extension for 1 min at 58°C; and post-PCR for 30 s at 25°C. The PCR system used was the StepOnePlus real-time PCR system (Applied Biosystems).

### Statistical analysis

Tests for Hardy-Weinberg equilibrium in the distribution of allelic frequencies among genotypes in this study population were carried out using the χ^2^ test, and a p-value of <0.05 was considered to represent a statistically significant difference. The association between the therapeutic response to IFX during maintenance therapy and genetic polymorphisms of the 8 single-nucleotide polymorphisms (SNPs) was evaluated using the multivariate logistic regression model, calculated odds ratios (ORs) for therapeutic response, and 95% confidence intervals (CIs). A stepwise backward-elimination procedure was used.

The crude OR in univariate analysis was calculated as the response to IFX during maintenance therapy based on patient characteristics (sex, body weight, age, disease duration, smoking history, IFX double dosing, with or without concomitant medications (5-ASA, IM), and each of the 8 SNPs).

For a multivariable logistic model, we used remission or nonremission as dependent variables, and sex, body weight, age, disease duration, smoking history, IFX double dosing, with or without concomitant medications (5-ASA, IM), and each SNP as independent variables. Variables with p<0.5 in univariate analysis were entered together into a multivariable logistic model and then removed until all retained variables had a value of p<0.1. Analysis was conducted including the following potential confounders: patient characteristics of sex, body weight, age, disease duration, smoking history, IFX double dosing, with or without concomitant medications (5-ASA, IM), and the 8 SNPs of TNF-α listed above.

For a final predictive model equation for the therapeutic response, we used the dependent variables that obtained statistical significant variables in a multivariable logistic model.

All statistical analyses were conducted using the IBM SPSS Statistics for Windows, version 23.0J (IBM SPSS Statistics, Chicago, IL, USA).

## Results

### Study population

A total of 121 Japanese CD patients were enrolled in this study. The patient characteristics are shown in Table 1. The mean (± SD) body weight was 62.5 ± 14.2 kg. The mean age was 37.5 ± 9.5 years, and 24.0% were women. The mean disease duration was 14.2 ± 7.8 years. The proportions of current smokers, past smokers, and nonsmokers were 14.9%, 7.4%, and 77.7%, respectively. 70.2% of the patients were receiving IFX at the dose of 5 mg/kg and 29.8% at the dose of 10 mg/kg. 5-ASA and IM were concomitantly administered to 66.9% and 48.8% of the patients, respectively. The mean CDAI score was 87.63 ± 59.76 and mean CRP level was 0.49 ± 0.96 mg/dL.

**Table 1:** Characteristics of the study population.

Patients (n)		121
Sex	Male, n (%)	92 (76.0)
	Female, n (%)	29 (24.0)
Body weight (kg)	Mean ± SD	62.5 ± 14.2
Age (yr)	Mean ± SD	37.5 ± 9.5
CD type classification	Ileum, n (%)	25 (20.7)
	Ileocolic, n (%)	75 (62.0)
	Colon, n (%)	19 (15.7)
	Unknown, n (%)	2 (1.6)
Disease duration (yr)	Mean ± SD	14.2 ± 7.8
Smoking	Current smoker, n (%)	18 (14.9)
	Past smoker, n (%)	9 (7.4)
	Nonsmoker, n (%)	94 (77.7)
Double dosing	5 mg/kg, n (%)	85 (70.2)
	10 mg/kg, n (%)	36 (29.8)
Combination treatment	5-ASA, n (%)	81 (66.9)
	IM, n (%)	59 (48.8)
CDAI score	Mean ± SD	87.63 ± 59.76
CRP level (mg/dL)	Mean ± SD	0.49 ± 0.96

5-ASA: 5-amino salicylic acid, IM: immunomodulator, CDAI: Crohn’s Disease Activity Index, CRP: C-reactive protein.

### Genotyping

Frequencies of each genotype and allele in the 121 patients are shown in Table 2. The frequencies of genotypes for TNF-α -238 G>A (rs361525) were not in agreement with Hardy-Weinberg equilibrium, although the frequencies of all other genotypes were.

**Table 2:** Genotype frequency and allele frequency in Japanese CD patients (n = 121).

Gene	Genotype frequency				Allele frequency		HWE^†^
TNF-α -238G>A (rs361525)	G/G	G/A	A/A	G/A or A/A	G	A	0.01
	114 (94.2)	6 (5.0)	1 (0.8)	7 (5.8)	234 (96.7)	8 (3.3)	
TNF-α -308G>A (rs1800629)	G/G	G/A	A/A	G/A or A/A	G	A	0.74
	114 (94.2)	7 (5.8)	0 (0.0)	7 (5.8)	235 (97.1)	7 (2.9)	
TNF-α -857C>T (rs1799724)	C/C	C/T	T/T	C/T or T/T	C	T	0.62
	81 (66.9)	35 (28.9)	5 (4.1)	40 (33.1)	197 (81.4)	45 (18.6)	
TNFR1 A>G (rs767455)	A/A	A/G	G/G	A/G or G/G	A	G	0.71
	96 (79.3)	24 (19.8)	1 (0.8)	25 (20.7)	216 (89.3)	26 (10.7)	
TNFR2 G>A (rs976881)	G/G	G/A	A/A	G/A or A/A	G	A	0.09
	89 (73.6)	32 (26.4)	0 (0.0)	32 (26.4)	210 (86.8)	32 (13.2)	
TNFR2 T>G (rs1061622)	T/T	T/G	G/G	T/G or G/G	T	G	0.47
	92 (76.0)	28 (23.1)	1 (0.8)	29 (24.0)	212 (87.6)	30 (12.4)	
FCGR2A A>G (rs1801274)	A/A	A/G	G/G	A/G or G/G	A	G	0.29
	71 (58.7)	46 (38.0)	4 (3.3)	50 (41.3)	188 (77.7)	54 (22.3)	
FCGR3A T>G (rs396991)	T/T	T/G	G/G	T/G or G/G	T	G	0.21
	62 (51.2)	53 (43.8)	6 (5.0)	59 (48.8)	177 (73.1)	65 (26.9)	

^†^HWE, Hardy-Weinberg equilibrium.

### Association beween therapeutic response to IFX in long-term continuous administration and genetic polymorphisms

Associations beween the therapeutic response to IFX during long-term maintenance treatment and genetic polymorphisms in the 121 Japanese CD patients are shown in Tables 3 and 4. Univariate analysis revealed that longer disease duration and double dosing of IFX were related to nonremission during maintenance therapy, although the presence of any SNP did not result in a statistically significant difference between the remission and nonremission groups.

**Table 3:** Association between IFX therapeutic response and patient characteristics.

		Remission (n = 71)	Nonremission (n = 50)	Crude OR (95% CI)	p-value	Adjusted OR (95% CI)^※1^	p-value
Sex	Men, n (%)	57 (80.3)	35 (70.0)	0.57 (0.25–1.33)	0.20	0.35 (0.11–1.19)	0.09
	Women, n (%)	14 (19.7)	15 (30.0)				
Body weight (kg)	Mean ± SD	62.5 ± 13.1	62.6 ± 15.7	1.00 (0.97–1.03)	0.95	0.97 (0.93–1.00)	0.08
Age (yr)	Mean ± SD	36.1 ± 10.3	39.6 ± 7.9	0.96 (0.92–1.00)	0.05	―	―
Disease duration (yr)	Mean ± SD	12.1 ± 7.5	17.3 ± 7.4	0.91 (0.87–0.96)	<0.01	0.90 (0.85–0.96)	<0.01
Smoking	Current, n (%)	9 (12.7)	9 (18.0)	0.76 (0.46–1.24)	0.27	0.63 (0.32–1.23)	0.19
	Past, n (%)	4 (5.6)	5 (10.0)				
	Nonsmoking, n (%)	58 (81.7)	36 (72.0)				
Double dosing	5 mg/kg, n (%)	61 (85.9)	24 (48.0)	0.15 (0.06–0.36)	<0.01	0.09 (0.03–0.28)	<0.01
	10 mg/kg, n (%)	10 (14.1)	26 (52.0)				
Combination treatment	5-ASA, n (%)	50 (70.4)	31 (62.0)	1.46 (0.68–3.14)	0.33	1.43 (0.53–3.80)	0.48
	IM, n (%)	39 (54.9)	20 (40.0)	1.83 (0.88–3.81)	0.11	4.45 (1.50–13.19)	<0.01
TNF-α -238G>A (rs361525)	G/G	66 (93.0)	48 (96.0)	1.82 (0.34–9.77)	0.49	―	―
	G/A or A/A	5 (7.0)	2 (4.0)				
TNF-α -308G>A (rs1800629)	G/G	67 (94.4)	47 (94.0)	0.94 (0.20–4.38)	0.93	―	―
	G/A or A/A	4 (5.6)	3 (6.0)				
TNF-α -857C>T (rs1799724)	C/C	50 (70.4)	31 (62.0)	0.69 (0.32–1.47)	0.33	0.33 (0.12–0.95)	0.04
	C/T or T/T	21 (29.6)	19 (38.0)				
TNFR1 A>G (rs767455)	A/A	57 (80.3)	39 (78.0)	0.87 (0.36–2.12)	0.76	―	―
	A/G or G/G	14 (19.7)	11 (22.0)				
TNFR2 G>A (rs976881)	G/G	52 (73.2)	37 (74.0)	1.04 (0.46–2.37)	0.93	―	―
	G/A or A/A	19 (26.8)	13 (26.0)				
TNFR2 T>G (rs1061622)	T/T	54 (76.1)	38 (76.0)	1.00 (0.43–2.33)	0.99	―	―
	T/G or G/G	17 (23.9)	12 (24.0)				
FCGR2A A>G (rs1801274)	A/A	40 (56.3)	31 (62.0)	1.26 (0.60–2.65)	0.53	―	―
	A/G or G/G	31 (43.7)	19 (38.0)				
FCGR3A T>G (rs396991)	T/T	37 (52.1)	25 (50.0)	0.92 (0.45–1.90)	0.82	―	―
	T/G or G/G	34 (47.9)	25 (50.0)				

^※1:^ Adjusted for sex, body weight, disease duration, smoking, double dosing, 5-ASA, IM.

**Table 4:** Logistic regression analysis of the four variables as predictive factors for IFX therapeutic response.

Independent variable	β	SE β	Wald χ^2^	p value	OR^a^	95%CI OR
Disease duration	-0.1050	0.0315	11.13	0.0009	0.035	0.005–0.252
Double dosing	-1.1413	0.2764	17.05	<0.0001	0.102	0.035–0.301
Combination treatment with IM	0.7265	0.2595	7.84	0.0051	4.276	1.546–11.826
TNF-α- 857C>T (C/C)^b^ (rs1799724)	-0.4160	0.2491	2.79	0.0949	0.435	0.164–1.155
Constant	1.3322	0.4976	7.17	0.0074		

^a^Odds ratio (OR) is given with 95%CI after using a logistic regression analysis of the four variables as predictive factors for IFX therapeutic response.

^b^The homozygous genotype with minor allele was combined to heterozygous genotype within parenthesis is indicates reference group.

Logit(*R*) = 1.3322 − 0.1050×Disease duration − 1.1413×Double dosing + 0.7265×Combination treatment with IM − 0.4160×TNF-α- 857C>T

*R*: Remission in IFX therapeutic response

For multicolinearity among each covariate in the patients, a correlation was seen between age and disease duration. Therefore, taking the results of univariate analysis and clinical significance into account, disease duration and age were selected as covariates for incorporation into multivariate analysis. A statistically significant difference was seen in multivariate analysis for disease duration, IFX double dosing, no concomitant use of an IM, and occurrence of TNF-α -857 C>T (rs1799724) as factors related to nonremission during IFX maintenance therapy.

## Discussion

To establish an individualized IFX treatment strategy in Japanese CD patients, we investigated the association between the therapeutic response during maintenance treatment and patient characteristics including genetic polymorphisms. Multivariate analysis identified longer disease duration, IFX double dosing (10 mg/kg), no concomitant use of an IM, and the TNF-α -857 C>T (rs1799724) polymorphism as factors related to nonremission during IFX maintenance therapy.

Patients with longer disease duration may be more likely to develop bowel complications such as strictures, making it more difficult to control CD with IFX. We also found that the therapeutic response to IFX during maintenance therapy was decreased in patients receiving a double dose (10 mg/kg) of IFX. Since double dosing is administered in patients who lose the response to the standard dose (5 mg/kg), this result indicates that patients might have become nonresponsive to TNF-α blockade, probably due to ATI production. Patients receiving IMs showed a better therapeutic response to IFX during maintenance therapy. This is consistent with a report demonstrating that combination therapy with IM was superior in terms of clinical efficacy compared with IFX monotherapy in patients with CD [25].

An association between the therapeutic response to IFX and genetic polymorphisms was reported in many investigations. We summarized the previous reports in Table 5 [15,16,18,19,25–28]. Those studies differed from ours in terms of evaluation timing and evaluation method as well as of patient ethnicity. Our study focused on the association between the therapeutic response to IFX during maintenance therapy and SNPs of several genes that were found to be associated with the response to IFX. Multivariate logistic analysis adjusted by patient characteristics showed that the therapeutic response to IFX was decreased in patients with the mutant (T) allele of TNF-α -857 C>T (rs1799724) compared with those with the wild-type allele (C/C) (OR = 0.33 95%, CI: 0.12–0.95). It was reported that the TNF-α -857T allele increases the transcription activity, mRNA expression, and protein expression of TNF-α compared with the wild-type C allele by 1.3-fold, 47.2-fold, and 3.4-fold, respectively [15]. Patients with the mutant TNF-α -857T allele might have higher production of TNF-α in the intestine and thereby show a decreased therapeutic response to IFX. The TNF-α -857 C>T (rs1799724) genetic polymorphism is considered to cause interindividual differences in the therapeutic response to IFX during maintenance therapy in Japanese patients with CD.

**Table 5:** Summary of the association between IFX treatment outcome and gene polymorphisms in various ethnic groups.

Gene	n	Disease	Population	IFX response^※1^	Response criteria	Follow-up period (weeks)	Adjustment^※2^	Reference No.
TNF-α -308G>A (rs1800629)	82	IBD	Spanish	↓A allele	HBI	4	No	[25]
	77	CD	Belgian	↑G Allele	CDAI	―	―	[19]
TNFR1 A>G (rs767455)	80	CD	Japanese	↓G Allele	HBI	4	Yes	[15]
	166	CD	Flemish	↓G Allele	CDAI, CRP	4	Yes	[27]
TNFR2 G>A (rs976881)	124	CD	Danish	↓A Allele	CRP	―	No	[28]
TNFR2 T>G (rs1061622)	124	CD	Danish	↑G Allele	CRP	―	No	[28]
	131	CD	Spanish	↑G Allele	HBI	10	Yes	[16]
FCGR3AT>G (rs396991)	102	CD	Japanese	↑GG genotype	CDAI, CRP	8, 30	No	[26]
	200	CD	Belgian	↑GG genotype	CDAI, CRP	4, 10	Yes	[18]

^※1:^ ↑Better response, ↓poorer response;

^※2:^ Adjusted for patient characteristics; HBI: Harvey-Bradshaw Index.

We did not find statistically significant differences in the association between the therapeutic response to IFX maintenance therapy and known genetic polymorphisms related to the IFX response (TNFR1 A>G [rs767455], TNFR2 G>A [rs976881], TNFR2 T>G [rs1061622], and FCGR3AT>G [rs396991]). These inconsistent results might be due to the evaluation timing (short or long term), evaluation index (Harvey-Bradshaw Index or CDAI), and/or ethnicity [16,17].

FCGR2A A>G (rs1801274) was analyzed because the association between this gene polymorphism and therapeutic response to IFX was reported in patients with rheumatoid arthritis [26,29]. A statistically significant association between this polymorphism and the response to IFX during maintenance therapy was not seen in this population of patients with CD. The therapeutic effect of IFX may therefore differ depending on disease pathophysiology.

### Study strengths and limitations

One of the strengths of this study is that we investigated the contribution of genetic polymorphisms to the therapeutic response to IFX maintenance therapy in Japanese patients with CD by considering factors potentially affecting the response and adjusting patient characteristics. As shown in Table 5, only a few previous studies adjusted patient characteristics, which allows appropriate, precise evaluation of the effects of genetic polymorphisms. In addition, we defined the therapeutic response by combining the CRP level, an objective inflammatory index, and the CDAI, a subjective disease activity index. In previous studies, only subjective indices such as the Harvey-Bradshaw Index and CDAI were used; even when both CDAI score and CRP level were used, they were evaluated separately. The therapeutic response was thus assessed more precisely in the present study. Furthermore, mean values of CDAI scores and CRP levels measured during 1-year follow-up in each patient were calculated, thereby avoiding the effect of transient aggravation due to causes other than CD, such as infection.

We aware several limitations of this study. First, the duration of treatment with IFX varied among patients. Longer treatment duration was associated with a decreased response to IFX, and the various disease durations may have affected the results. Another limitation is that we did not measure ATI and trough levels of IFX. It is known that the presence of ATI and/or lower trough levels of IFX are associated with a reduced response to IFX.

## Conclusions

We showed that remission during IFX maintenance therapy in Japanese CD patients was associated with the TNF-α 857C>T C/C genotype, shorter disease duration, no double dosing, and combination treatment with an IM. These results could potentially be utilized to establish an individualized IFX treatment strategy for Japanese or other CD patients.

## Supporting information

S1 Table The raw data of the study. (CSV)
